# Can inactivation mutation in the thyroid stimulating hormone receptor gene and hyperthyroidism coexist?: A case report

**DOI:** 10.1097/MD.0000000000036950

**Published:** 2024-01-19

**Authors:** Yanfang Liu, Jie Li, Fei Gao, Changjian Zhao, Luyang Yang, Yunfeng Liu

**Affiliations:** a Department of Endocrinology, First Hospital of Shanxi Medical University, Taiyuan, China; b Department of Urology, First Hospital of Shanxi Medical University, Taiyuan, China.

**Keywords:** inactivation mutation, monoallelic heterozygous mutation, TSHR gene

## Abstract

**Introduction:**

We found the G132R heterozygous mutation of thyroid stimulating hormone receptor (TSHR) gene in a patient with recurrent hypokalemia. Because the patient had a medical history of hyperthyroidism, the mutation was suspected to be related to hyperthyroidism at first. Subsequently, the expression and function studies in vitro were conducted.

**Methods:**

Wide-type TSHR and mutant TSHR (mutTSHR) were constructed in the phage vector and pEGFP-C1 vector. After transfection, the samples were collected for detection of mRNA level, protein expression, cell activity and cAMP content.

**Results:**

Compared with the wild-type TSHR, the mRNA level of the mutTSHR was not significantly different. But the protein expression, cell activity and cAMP content of the mutTSHR were significantly lower. So this indicated that the G132R mutation is a loss-of-function mutation.

**Conclusion:**

We identified the G132R monoallelic heterozygous mutation of TSHR gene in a patient with hyperthyroidism. Based on disease history of the patient, we speculated that the heterozygous mutation did not cause thyroid dysplasia or hypothyroidism for her. Our study enriched experiment content in vitro studies and clinical phenotype about the G132R mutation in TSHR gene.

## 1. Introduction

Thyroid stimulating hormone receptor (TSHR) can affect the proliferation, differentiation and function of thyroid gland by promoting adenosine cyclase (AC)-cAMP and phospholipase C-inositol 1,4,5-triphosphate-Ca^2+^ signaling pathways under the stimulation of thyroid stimulating hormone (TSH).^[1]^ TSHR is encoded by TSHR gene. When its gene mutates, any link in the cascade reaction chain may change, leading to related thyroid diseases. TSHR gene activating mutation may cause thyroid hyperfunction adenoma, non-autoimmune hyperthyroidism, and toxic multinodular goiters.^[2]^ Inactivated mutations in TSHR gene may result in thyroid dysplasia and different degrees of TSH resistance.^[3]^

It is difficult to link the inactivated mutation to hyperthyroidism. Here, we report a patient of inactivation mutation in TSHR gene complicated with hyperthyroidism, which improve clinicians’ understanding of TSHR gene mutation and provide diagnosis experience.

## 2. Patients and clinical characteristics

The patient was a 60-year-old female with a 1-year history of type 2 diabetes and hyperthyroidism, which was treated with antidiabetic and antithyroid drugs regularly, such as metformin (1.5 g/d) and methimaole (10 mg/d). In addition, the patient received left adrenal adenoma resection due to Cushing syndrome (CS) before 8 months, and she need to take prednisone (20 mg/d). The patient was admitted to our hospital with symptoms of fatigue, nausea and anorexia, and then received a detailed endocrine assessment, including plasma cortisol, plasma renin activity and aldosterone, thyroid function, blood gas analysis, electrolytes, thyroid ultrasound, etc (shown in Table 1). The results suggested that her serum potassium level were below normal. And her cortisol and plasma aldosterone levels were also low. Further, her hyperthyroidism seems to have worsened. After making a detailed inquiry about her medical history, we found that the patient did not take hormone substitutes and antithyroid drugs regularly. So, we prescribed the patient hydrocortisone (the initial dose was 40 mg/d, subsequently the dose reduced to 10 mg/d.) and methimazole (20 mg/d). And we noticed during the admission period, her serum potassium level was still below 2.8 mmol/L despite the daily supplement of 3 g of potassium chloride.

**Table 1:** The laboratory tests in our hospital.

Laboratory tests	Measured value	Normal range
Hormones		
Cortisol at 0 am (nmol/L)	2.8	–
Cortisol at 8 am (nmol/L)	5.4	171–536
Cortisol at 4 pm (nmol/L)	16.10	64–327
ACTH (pmol/L)	0.71	1.6–13.9
24h UFC (nmol/24 h)	10.83	100–379
Plasma renin activity recumbent (ng/mL/h)	0.73	0.05–0.79
Aldosterone recumbent (pg/mL)	26.86	59–174
Plasma renin activity upright (ng/mL/h)	6.22	0.93–6.56
Aldosterone upright (pg/mL)	45.41	65–296
ARR upright	7.3	–
Thyroid function		
FT3 (pmol/L)	26.96	3.1–6.8
FT4 (pmol/L)	72.02	10–23
TSH (uIU/mL)	0.005	0.27–4.2
TPOAb (IU/mL)	9.27	0–34
TGAb (IU/mL)	18.23	0–115
Blood gases		
PH	7.454	7.35–7.45
PCO_2_ (mm Hg)	35.20	35–45
HCO3^-^ (mmol/L)	24.30	21–26
Else		
K^+^ (mmol/L)	2.64	3.5–5.5
HbA1c (%)	7.0	4.8–5.9

ACTH = adrenocorticotropic hormone, ARR = aldosterone-to-renin ratio, FT3 = free triiodothyronine, FT4 = free thyroxine, HbA1c = glycosylated hemoglobin, PH = pondus hydrogenii, TGAb = thyroglobulin antibodies, TPOAb = thyroperoxidase antibodies, TSH = thyroid stimulating hormone, UFC = urinary free cortisol.

## 3. Gene sequencing

**Figure 1.**
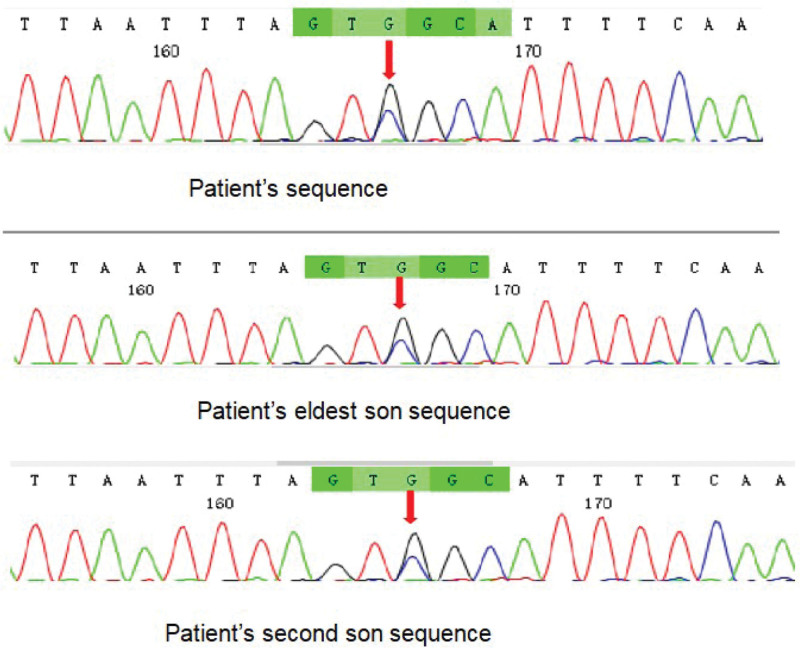
TSHR gene fragment sequence containing the mutation exon5:c.G394C:p.G132R in the patient and her 2 sons. The base substitution mutation is indicated by a red arrow. At nucleotide position 394, a heterozygous guanine-to-cytosine point mutation was identified, resulting in a substitutions of arginine (CGC) in place of glycine (GGC) at codon 132.

To further clarify the cause of hypokalemia, with the patient consent, her blood samples were sent to Shanghai Weihan company for sequencing. Genomic DNA was extracted from the samples of the subjects and a genomic library was constructed. The exons of the target gene and the adjacent splice region (about 50 bp) were captured by probe hybridization and enriched. The enriched genes were quality controlled and sequenced by illumina HiSeqX. After detection and analysis, a heterozygous missense mutation c.G394C:p.G132R was found in exon 5 of TSHR gene. Subsequently, we obtained blood samples from her 2 sons, both of them had the same mutation with their mother. The sequencing chromatogram of the patient and her sons are shown in Figure 1. Because the patient had a history of hyperthyroidism, we considered whether the gene mutation was related to hyperthyroidism, so we conducted vitro tests to verify the expression and function of the mutant TSHR (mutTSHR) compared to the wide-type TSHR (wtTSHR).

## 4. Expression and function in vitro studies

### 4.1. Vectors construction and cells transfection

The TSHR cDNA was prepared. Then phage-TSHR-SalI-F and phage-TSHR-NotI-R were used as primers to amplify the phage-TSHR-wt fragment. And, pEGFP-C1-TSHR-EcoRI-F and pEGFP-C1-TSHR-KpnI-R were used as primers to amplify the pEGFP-C1-TSHR-wt fragment. Similarly, phage-TSHR-wt fragment and pEGFP-C1-TSHR-mut fragment were obtained by site-specific mutagenesis of the primer TSHR-mut-F and TSH-mut-R. Finally, wtTSHR and mutTSHR were constructed in the phage vector and pEGFP-C1 vector.

293T cells were cultured in DMEM containing 10% fetal calf serum. The constructed eukaryotic expression vectors of wtTSHR and mutTSHR were transiently transfected into 293T cells constructed by Lipofectamine reagent according to the manufacturer instructions. The amount of transfected DNA was 1.5 µg/well.

### 4.2. Detection of mRNA level

After 48 hours of transfection, cells samples were collected. Total RNA was extracted by Trizol method, and cDNA synthesis was carried out after DNA digestion. The expression difference in the target genes of wtTSHR and mutTSHR was detected by quantitative real-time PCR method.

### 4.3. Western blot (WB) analysis

After 48 hours of transfection, cells precipitation were collected. RIPA lysate was used to extract the total protein in the cells precipitation. BSA kit was used to measured the protein concentration. Subsequently, the proteins were purified by denaturation, and WB method was used to detect the expression difference in the target proteins of the wtTSHR and mutTSHR.

### 4.4. Detection of cell proliferation using Cell Counting Kit-8

The cells were digested and collected respectively after 24 and 48 hours. After resuspension, the cell density was adjusted to 105 cells/mL. 100 µL cell suspension was added to each well of 96 well plate. 10 µL Cell Counting Kit-8 was added to each well after 5% CO_2_ culture overnight at 37 °C, and the cell proliferation activity was detected at 450 nm after incubation at 37 °C for 1 hour.

### 4.5. Detection of cAMP content

Due to the lack of reagents in our country, we couldn’t analyze the TSH-stimulated cAMP production. Therefore, after 48 hours of transfection, cell precipitation and culture medium supernatant samples were collected respectively. After ultrasound, centrifugation and deprecipitation, cAMP was measured by cAMP ELISA kit (GE Healthcare) according to the manufacturer protocol.

## 5. Statistical analysis

All data were analyzed and plotted using GraphPad Prism 8. Unless otherwise stated, all values were expressed as mean ± standard error of mean. Statistical analysis between the wtTSHR and mutTSHR groups was performed using Student *t* test. *P* < .05 was considered to represent a statistically significant difference.

## 6. Results

### 6.1. Recombinant vector sequencing

**Figure 2.**
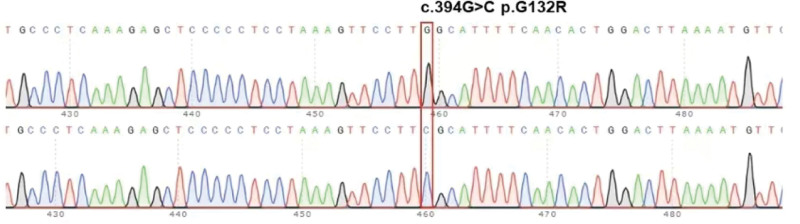
Sequencing result showed the mutation c.394G > C p.G132R was constructed successfully.

The mutation c.394G > C p.G132R construct was verified by means of Sanger sequencing (Fig. 2).

### 6.2. mRNA expression

**Figure 3.**
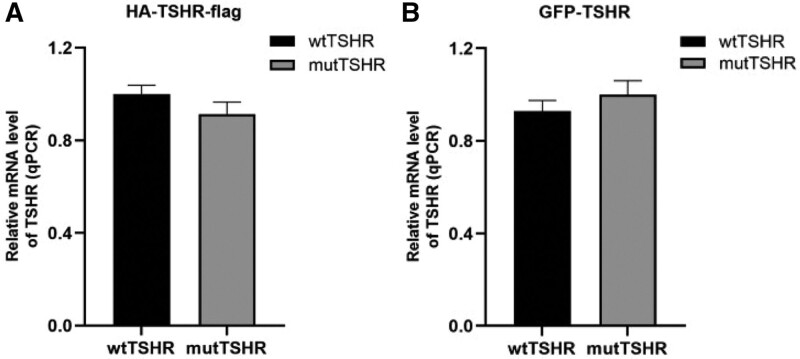
Detection of mRNA level of TSHR using qPCR. (A) mRNA level of TSHR in the phage vector. (B) mRNA level of TSHR in the pEGFP vector. mutTSHR = mutant TSHR, qPCR = quantitative real-time PCR, wtTSHR = wide-type TSHR.

In 293T cells, the expression results of the 2 sets of vectors showed that there were no significant difference in the mRNA expression at the transcriptional level between the wtTSHR and mutTSHR (Fig. 3).

### 6.3. Protein expression

TSHR protein contains the a and b subunit, linked by a C peptide and a disulfide bond, which dissociate after proteolytic hydrolysis. The a subunit is the extracellular segment encoded by exons 1 to 9. The b subunit is encoded by exon 10 containing some of the linker peptide and a transmembrane region. This mutation occurs within the a subunit.

**Figure 4.**
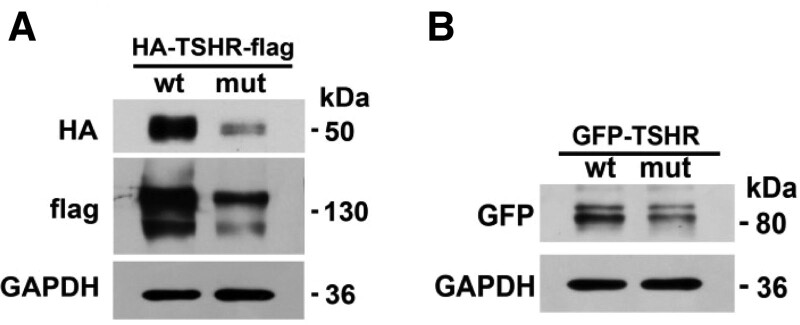
Detection of TSHR protein expression. (A) The fusion protein HA-TSHR-Flag was expressed in the phage vector. (B) The fusion protein GFP-TSHR was expressed in the pEGFP-C1vector. mut = mutant, wt = wide type.

In the phage vector, the fusion protein HA-TSHR-Flag was expressed, and HA and Flag tags were attached on both sides of the target protein. Compared with the GAPDH (the GADPH gene is expressed at high levels in almost all tissues and is widely used as internal reference for protein standardization in WB detection), detection results of anti-HA showed that a subunit band (−50 kDa) decreased significantly. Similarly, test results of anti-Flag suggested that the full-length protein product (120–130 kDa) after mutation was significantly decreased (Fig. 4A).

In the pEGFP-C1 vector, the fusion protein GFP-TSHR was expressed, and the label GFP was at the N-terminal of the protein. Detection results of anti-GFP revealed that the a subunit band (−80 kDa) was significantly decreases compared to GADPH (Fig. 4B).

In a word, the protein expression of mutTSHR significantly lower compared to the wtTSHR, indicating that the TSHR gene after mutation was inhibited at both the transcription and translation levels.

## 7. Cell viability

**Figure 5.**
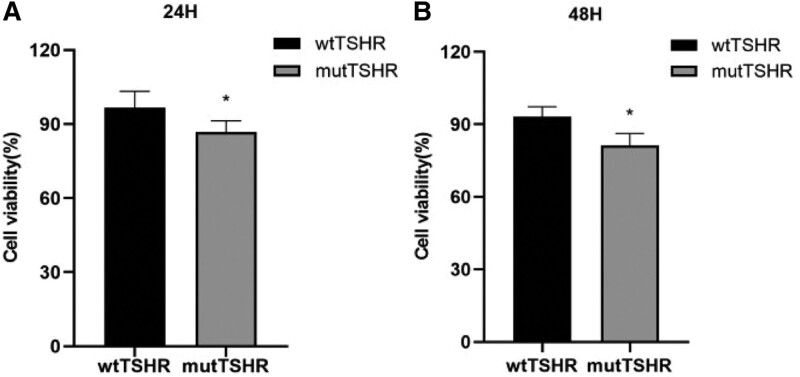
Detection of cell proliferation using CCK8. (A) Comparison of cell viability between wtTSHR cells and mutTSHR cells after 24-h transfection. (B) Comparison of cell viability between wtTSHR cells and mutTSHR cells after 48-h transfection. ^*^ denotes *P* < .05. CCK8 = Cell Counting Kit-8, mutTSHR, mutant TSHR, wtTSHR, wide-type TSHR.

Compared with the wtTSHR cells, detection results showed that the mutTSHR cells could significantly reduce activity (Fig. 5).

## 8. cAMP content

**Figure 6.**
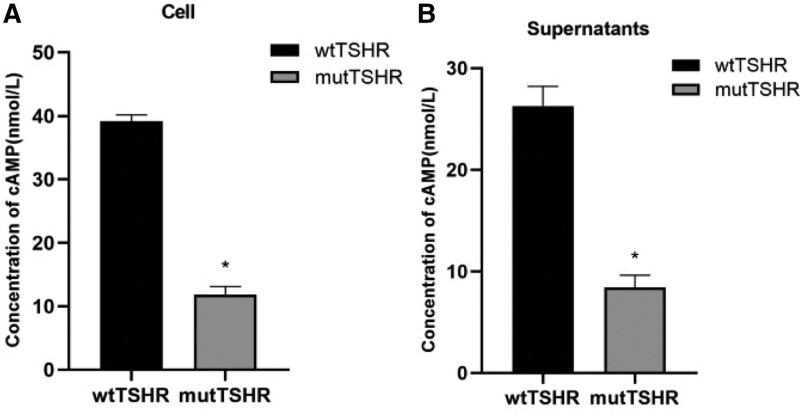
Detection of cAMP content. (A) Comparison of cAMP content between wtTSHR and mutTSHR in the cell. (B) Comparison of cAMP content between wtTSHR and mutTSHR in the supernatants. ^*^ denotes *P* < .05. mutTSHR = mutant TSHR, wtTSHR = wide-type TSHR.

Compared with the wtTSHR cells, detection of ELISA indicated that the mutTSHR cells could significantly reduce the cAMP content of intracellular and extracellular (Fig. 6).

## 9. Treatment

After receiving genetic analysis results, we excluded the rare genetic diseases that lead to hypokalemia such as Liddle syndrome, mineralocorticoid excess. And then we found that even through the patient had take antithyroid drugs adequately, her thyroid function was still poorly control. We speculated that this low serum potassium is related to the deterioration of hyperthyroidism, so it was suggested that the patient receive radioactive iodine therapy. The patient was followed up at 3 and 6 months after radioactive iodine therapy. The follow-up results of her potassium, free triiodothyronine, and free thyroxine levels before and after treatment are shown in Table 2. Following treatment, the patient’ s thyroid function had improved, and her serum potassium level was within the normal range.

**Table 2:** Serum potassium levels and thyroid function during the clinical course of the patient.

Time	Serum potassium (mmol/L)	FT3 (pmol/L)	FT4 (pmol/L)	TSH (uIU/mL)
First admission	2.64↓	26.96↑	72.02↑	0.005↓
Three mo after radioiodine therapy	4.42	10.20	18.75↑	0.008↓
Six mo after radioiodine therapy	4.53	5.1	12.27	0.008↓

↓ indicates that the value is less than the normal range; ↑ indicates that the value is out of the normal range.

## 10. Discussion

At first, the patient suffered from weakness. After examination and treatment, repeated hypokalemia was found. According to the results of the patient laboratory tests, first of all, the low levels of cortisol and adrenocorticotropic hormone suggested that hypokalemia caused by CS may be ruled out. In addition, low plasma aldosterone levels and normal blood pressure showed that hypokalemia resulted from primary or secondary aldosteronism could be excluded. Furthermore, because her arterial blood gas and urine analysis, we did not consider renal tubular acidosis or Fanconi syndrome as the possible cause. We were puzzled by the repeated hypokalemia, which continued to recur even after she received daily potassium supplementation (3 g of potassium chloride per day). Therefore, the patient blood samples were sent for sequencing in order to further clarify the cause of hypokalemia. Genetic analysis results showed that the patient had a heterozygous missense mutation in the TSHR gene: exon 5:c.G394C:p.G132R. The heterozygous mutation resulted in arginine substituted glycine in exon 5 of TSHR gene. Then, the same mutation was screened for her 2 sons, and it was found that both sons had the same heterozygous mutation.

TSHR gene is located in chromosome 14q31, with a length of 60 kb. It has 10 exons and 9 introns. According to the 3-dimensional tissue model, it is divided into 3 parts: extramembranous region, transmembrane region and intramembrane region.^[4]^ The extracellular domain is encoded by exons 1 to 9, which is related to the glycosylation of TSHR, the site of mutual recognition and action of hormone and ligands. The transmembrane and intramembrane regions are encoded by exon 10 and are important sites for transmitting signals to cells. TSHR belongs to the 7-fold transmembrane G protein coupled receptor family, which regulates thyroid hormone secretion and thyroid cells growth through cAMP pathway mediated by G protein. In addition, thyroid iodization and thyroid hormone synthesis can be regulated by phospholipase C pathway.^[1]^ Therefore, once the TSHR gene mutated, function of TSHR protein including the expression level on the plasma membrane, signal transmission of receptors, and combination with ligands could be damaged. TSHR gene mutations include activation mutations and inactivation mutations. In the absence of ligands binding, TSHR activation mutations could autonomously activate AC-cAMP pathway, leading to proliferation and hyperfunction of thyroid cells, thereby causing autonomic hyperfunctional thyroid adenoma, non-autoimmune hyperthyroidism, etc.^[2]^ The TSHR loss-of-function mutations are inactivation mutations, which leads to the reduction of TSHR function such as the expression of mut receptor on eukaryotic cell membrane, the ability of binding to TSH and the activity of activating AC, thus causing hyperthyroxemia and congenital hypothyroidism.^[3]^

The G132R mutation of TSHR gene in our paper occurs in exon 5, and its frequency of East Asian population in the gnomad database is about 0.06%. The results of bioinformatics software analysis suggested that this gene mutation may have harmful effects. The patient had a history of hyperthyroidism, we doubted whether the gene mutation may be related to hyperthyroidism. In order to verify the expression and function of the gene mutation, we conducted in vitro tests. The results of expression in vitro studies showed that compared with the wtTSHR, the mRNA level of the mutTSHR did not change significantly, but the expression level of protein decreased significantly. In addition, the functional tests results suggested that the heterozygous mutation leads to significantly decreased cell activity, cAMP production. The above manifested that the G132R mutation in TSHR gene is an inactivation mutation. It is consistent with the previous report,^[5]^ but vitro tests in our paper were much richer.

More than 40 kinds of TSHR inactivation mutations have been found in patients of different ethnic backgrounds and geographical areas, including missense mutations, nonsense mutations, deletion and insertion mutations, and most of the mutations occurred in exon 10. Some hot spot mutations (P162A, W546X, R450H, A553T, etc) were often reported.^[4,6]^ The inactivation mutation of TSHR gene has a wide range of clinical and serological manifestations, ranging from severe non-autoimmune hypothyroidism to hyperthyroxemia with normal thyroid function.^[7,8]^ The degree of TSH resistance depends on the severity of receptor function damage caused by mutation and the number of mut alleles.^[9]^ Previous reports have shown that carriers of homozygous or compound heterozygous mutations are more likely to develop congenital hypothyroidism, while subjects with monoallelic heterozygous mutation in the TSHR gene may present with normal thyroid function or subclinical hypothyroidism.^[5,10]^ According to numerous studies results, 2 modes of inheritance of TSHR inactivation mutations causing diseases are identified including autosomal recessive and autosomal dominant inheritance.^[11–13]^ Previous study found that the G132R mutation would reduce cAMP production and binding activity, and the patient had normal thyroid ultrasound and increased TSH level.^[14]^ In our paper, the results in vitro studies showed that the heterozygous mutation is loss of function mutation. And, the maximum cAMP production of the mutTSHR is less than half of that in the wtTSHR group, which suggested the loss of function of the mut is more than 50%. On the basis on the medical history and thyroid ultrasound findings of the patient, the heterozygous mutation did not cause thyroid dysplasia or hypothyroidism. And her 2 sons with the same heterozygous mutation had no thyroid dysfunction. It is probably because thyroid function and development are also affected by other genes. In addition, the degree of impaired TSHR function or thyroid hormone requirement in carriers with heterozygous gene mutation may change with age and other factors. This may help explain why these patients exhibited a variety of phenotypes ranging from normal thyroid function to mild hypothyroidism.^[15]^

As for the patient repeated hypokalemia, we finally considered that it was related to the patient poor control of hyperthyroidism after a series of exclusion for diseases of adrenal glands and kidneys. Hypokalemia due to hyperthyroidism is less common in women. Since the effect of anti-thyroid drugs was disappointing, the patient was treated with radioactive iodine in the later stage. According to the follow-up results, her thyroid function was improved, and the serum potassium level of the patient was within the normal range. Since the patient will have hypothyroidism after receiving radioactive iodine treatment,^[16,17]^ and the presence of heterozygous inactivation mutations may increase the risk of hypothyroidism in the future, the patient should pay more attention to the follow-up of thyroid function changes.

## 11. Conclusion

In conclusion, we have identified a TSHR inactivation missense mutation in a patient with hyperthyroidism. Our study not only adds experimental evidence for the G132R mutation in TSHR gene, but also provides valuable information for clinicians to interpret the significance of the inactivation mutation in TSHR gene.

## Acknowledgments

The authors express their gratitude to the patient and his family for their participation in this study. The authors are grateful to WE·HEALTH Biomedical Technology Co. Ltd., Shanghai, China, for the genetic analysis.

## Author contributions

**Data curation:** Yanfang Liu, Jie Li, Yunfeng Liu.

**Formal analysis:** Yanfang Liu, Jie Li, Fei Gao, Changjian Zhao, Luyang Yang, Yunfeng Liu.

**Funding acquisition:** Yunfeng Liu.

**Investigation:** Yanfang Liu, Jie Li, Fei Gao, Changjian Zhao, Luyang Yang, Yunfeng Liu.

**Methodology:** Yanfang Liu, Luyang Yang, Yunfeng Liu.

**Project administration:** Yunfeng Liu.

**Supervision:** Yunfeng Liu.

**Writing – original draft:** Yanfang Liu, Jie Li.

**Writing – review & editing:** Yanfang Liu, Jie Li, Yunfeng Liu.
